# Human differentiated eosinophils release IL-13 in response to IL-33 stimulation

**DOI:** 10.3389/fimmu.2022.946643

**Published:** 2022-09-13

**Authors:** Amiko M. Uchida, Gabrielle Ro, Li Qiang, Kathryn A. Peterson, June Round, Michael Dougan, Stephanie K. Dougan

**Affiliations:** ^1^ Division of Gastroenterology, Hepatology and Nutrition, University of Utah, Salt Lake City, UT, United States; ^2^ Cancer Immunology and Virology, Dana Farber Cancer Institute, Boston, MA, United States; ^3^ Gastrointestinal Unit, Department of Medicine, Massachusetts General Hospital, Boston, MA, United States; ^4^ Department of Medicine, Harvard Medical School, Boston, MA, United States; ^5^ Department of Pathology, University of Utah, Salt Lake City, Utah, United States; ^6^ Department of Immunology, Harvard Medical School, Boston, MA, United States

**Keywords:** atopy, allergy, eosinophilic esophagitis, type 2 inflammation, CD34 cells+

## Abstract

**Objective:**

Eosinophils are hallmarks in allergic type 2 inflammation and are known to release cytotoxic granule proteins that contribute to inflammation. Eosinophils develop in the bone marrow from hematopoietic stem cells and once mature, have a limited lifespan in culture, making them difficult to study *ex vivo*. IL-33 has increasingly been shown as a key regulator of type 2 inflammation *via* signaling through its receptor, ST2. The present study was conducted to detail a method of eosinophil differentiation from hematopoietic stem cells and determine the response to IL-33.

**Methods:**

CD34+ and CD14+ cells were isolated from donor apheresis cones and differentiated into eosinophils or macrophage controls, respectively. Morphologic, transcriptional and protein analyses were performed to validate this method of eosinophil differentiation. The effect of IL-33 on differentiated eosinophils was assessed using qPCR, immunofluorescence, and multiplex cytokine array.

**Results:**

CD34 differentiated eosinophils appear morphologically similar by H&E and express eosinophil peroxidase (EPX) protein as well as the conventional eosinophil transcripts *EPX*, *CLC*, and *MBP*. In addition, differentiated eosinophils expressed both isoforms of the IL-33 receptor, *ST2L* and s*ST2* throughout the differentiation process. Transcript levels of both IL-33 receptors were up-regulated by treatment with IL-33 at earlier timepoints in the differentiation. These cells also expressed *IL-4* and *IL-13* mRNA which were up-regulated by IL-33 as well. Notably, *IL-13* expression was significantly higher with IL-33 treatment compared to media control at every timepoint measured. IL-33 significantly increased cellular secretion of IL-13 protein at most timepoints throughout differentiation. IL-8, LIF, CCL1, CCL5, CCL7, and CCL8 were also significantly secreted after IL-33 stimulation.

**Conclusions:**

Our findings suggest that CD34 differentiated eosinophils are morphologically and phenotypically similar to peripheral eosinophils. The release of specific cytokines in direct response to IL-33 may contribute to the pathogenesis of type 2 inflammation and facilitates new avenues for studying eosinophils as effector cells *in vitro*.

## Introduction

Eosinophils are important innate immune effector cells in allergic responses (1). However, much remains unknown about these enigmatic cells including potential activation signals relevant to eosinophilic esophagitis (EoE). EoE is an increasingly common chronic allergic disease of the esophagus that typically presents with dysphagia and food impactions, and histologically is defined by elevated eosinophils in the esophagus, where they are normally absent (2). When activated, eosinophils degranulate and release nonenzymatic cationic proteins, among other effector molecules, that can lead to inflammation and damage the surrounding microenvironment (1).

We recently showed that tissue resident eosinophils from patients with EoE have high expression of the IL-33 receptor, ST2 (3). IL-33 is a member of the IL-1 superfamily of inflammatory cytokines and has been shown to be important and elevated in allergic diseases such as EoE, asthma and atopic dermatitis (4–6). IL-33 is elevated in the esophagus of patients with EoE (4) and can be released from various cell types including epithelial, endothelial and dendritic cells (5, 7, 8). ST2 has two isoforms (9); ST2L is membrane bound whereby upon engagement of IL-33, conventionally leads to downstream release of type 2 cytokines IL-5 and IL-13 by group 2 innate lymphoid cells (ILC2s) (8). Conversely, alternative splicing leads to expression of soluble ST2 (sST2), which acts as a decoy receptor limiting the bioactivity of IL-33. Importantly, type 2 inflammatory cytokines such as IL-4 and IL-13 are implicated in allergy and EoE. Selective blockade of these cytokines results in resolution of esophageal eosinophils and symptom resolution when IL-4 and IL-13 are collectively inhibited (10–12).

Taken together, these data suggest IL-33 and downstream type 2 cytokines IL-4 and IL-13 are important in EoE. However, whether eosinophils directly respond to IL-33 and release these type 2 cytokines has not been previously explored. Given the challenges of culturing mature eosinophils long-term *ex vivo*, here we expand upon prior reports of eosinophil differentiation from hematopoietic CD34+ stem cells and detail a protocol for this assay. This allows for *in vitro* characterization and manipulation of eosinophils (13, 14). We monitor cells across the differentiation process and show that differentiated eosinophils robustly release IL-13 in response to IL-33 stimulation, in addition to several other cytokines.

## Methods

### Human donor specimens

We utilized deidentified leukapheresis cones from Kraft Blood Donor Center at Dana Farber Cancer Institute Blood Bank and Associated Regional and University Pathologists (ARUP) at the University of Utah. Blood samples were donated without knowledge to these investigators as to the indication of donation. Donors did not receive mobilization therapies such as G-CSF. Samples were collected using a Terumo Trima Accel machine, Trima V.7 software and Trima Accel V.7 kits. These blood donor samples are exempt from IRB approval. Donors A-D were used for protein analyses as below, and Donors E-I were used for transcript experiments.

### Cell culture

Donor blood was diluted with one-part sterile PBS and one-part Ficoll-Paque (Cytiva) underlay. The suspension was centrifuged at 400xg for 30 minutes, no break, no acceleration. Peripheral blood mononuclear cells (PBMCs) were isolated from the buffy coat layer and washed with sterile PBS. CD34 positive selection was performed using a bead enrichment kit according to manufacturer protocol (Miltenyi) or stem cell technologies (Vancouver, Canada). The remaining CD34- cells underwent a positive selection for CD14 according to manufacturer protocol (Miltenyi). Mature eosinophils were isolated from donor samples using bead enrichment per manufacturer protocol (EasySep, Stem Cell Technologies).

Once enriched, CD34+ cells were then centrifuged and resuspended in Iscove’s modified Dulbecco’s medium (ATCC) containing 10% inactivated fetal calf serum (Peak Serum), 50 U/mL Penicillin-Streptavidin (Corning). On day 0, the cells were cultured at 500k/mL in this growth media supplemented with 50 ng/mL SCF, 10 ng/mL IL-6, 25 ng/mL IL-3, and 10 ng/mL GM-CSF (PeproTech). The media was replenished with these same cytokines on day 2 by adding one volume of growth media to the well to support expansion. Starting from day 5, half of the media was replaced every two to three days with growth media containing 25 ng/mL IL-3, 10 ng/mL GM-CSF, and 10 ng/mL IL-5 (PeproTech) until day 28.

CD14+ cells were centrifuged and resuspended in RPMI-1640 (Corning) containing 10% inactivated fetal calf serum (Gibco), 2 mM glutamine (Corning), 50 U/mL Penicillin-Streptavidin (Corning), and 1X Non-Essential Amino Acids (Corning). This growth media was supplemented with 50 ng/mL M-CSF (PeproTech) and the cells were plated at a density of 1 million/mL in 14cm petri dishes (Corning). Two days after the isolation, the cells were fed with more growth media containing 50 ng/mL M-CSF. On day 5, the CD14+ macrophage progenitors were largely adherent to the plates, therefore the cells were washed and detached with trypsin (Gibco) and replated at a density of 500k cells/mL in growth media with 50 ng/mL M-CSF.

To assess the effect of IL-33 on eosinophils, cells were stimulated with 100 ng/mL of IL-33 (PeproTech) at denoted timepoints and subsequently harvested 24 hours or 72 hours later for imaging, RNA, and cytokine analyses. CD14+ cells fully differentiate into macrophages by day 7, therefore these cells underwent one stimulation on day 6. Both cell populations were counted and plated at a concentration of 500k cells/mL.

### Histology and immunofluorescence

Differentiated eosinophils at designated timepoints were isolated at a concentration of 100k cells/100µL and centrifuged with a cytospin onto glass slides. Cells were fixed with 100% methanol for 60 seconds prior to hematoxylin and eosin staining (Abcam, ab246824) according to manufacturer protocol. Differentiated macrophages were grown on sterile glass coverslips in 6 well plates. Cells were fixed with 0.1 M MgSO4, 0.5 M PIPES, 0.1 M EGTA, 10% formaldehyde (Tousimis Research Corporation), all from Sigma, diluted with DPBS for 20 minutes at room temperature. Cells were permeabilized with 0.1% triton X for 2 minutes before blocking with Background Buster (Innovex or goat serum) for 1.5 hours.

A “no primary antibody” control was performed for every experiment and sample. Immunofluorescence staining panels were performed with appropriate primary and secondary antibody overnight at 4C or for 1 hour at room temperature for IL-13. Antibodies used: mouse-anti-human eosinophil peroxidase (EPX) (graciously provided by Dr. Elizabeth Jacobsen; Mayo Clinic, Scottsdale, AZ; mouse-anti-human, 1:200), goat-anti-mouse AF488 (BioLegend; Poly4054; 1:200), rat anti-human IL-13 (BioLegend; JES10-5A2; 1:200), mouse-anti-human IL-4 (Abcam, 5B5) conjugated to AF594 (Abcam ab269822; 1:200). Macrophages were stained with Phalloidin according to manufacturer protocol (Abcam ab176759). All cells were stained with DAPI nuclear dye for 5 minutes at room temperature. Images were obtained on a Nikon Ti inverted microscope or the Olympus IX83 multi-alkali PMT (x4) Spectral Imaging microscope at 20X if not otherwise noted. ImageJ/Fiji and Photoshop were used to interpret and display images.

### Flow cytometry

Differentiated eosinophils were stained using antibodies CCR3 (BioLegend clone 5E8, PE), Siglec-8 (BioLegend clone 7C9, PE/Dazzle), IL5-Ra (R&D Systems FAB253P, PE used in separate staining panel), CD11b (BioLegend clone M1/70 FITC), CD117 (BioLegend clone 104D2, BV421). Samples were run on using LSR Fortessa (BD Biosciences).

### Cytokine analysis

To measure IL-4 and IL-13 at varied timepoints after a stimulation, supernatants from cell concentration-controlled wells (500k cells/mL) were collected. Samples were sent for Luminex multiplex cytokine analysis to Eve Technologies (Calgary, Canada).

### qRT-PCR

Total RNA was isolated from cells using RNeasy Plus Mini Kits (Qiagen). RT was performed using the SuperScript™ IV First-Strand Synthesis System (Invitrogen). The expression levels of *EPX, CLC*, and *MBP* were measured to assess for presence of canonical eosinophil markers in differentiated cells. *IL-4, IL-13, sST2*, and *ST2L* were measured to examine downstream IL-33 transcriptional activity. *GAPDH* was used as the reference control sequence. The primers were obtained from IDT and the sequences are as follows: *CLC:* forward GGATGGCCAAGAATTTGAACTG, reverse GGTGTAAGAGGATTGGCCATTG (15); *EPX*: forward GTCCTGCGAGACTGCATAGC, reverse TATAATCTGCGGCCCGAACAA (PrimerBank ID 260064070c1); *MBP*: forward AAACTCCCCTTACTTCTGGC, reverse GCAGCGTCTTAGCACCCAA (PrimerBank ID 46276888c1); *IL-4*: forward CGGCAACTTTGTCCACGGA, reverse TCTGTTACGGTCAACTCGGTG (PrimerBank ID 4504669a2); *IL-13*: forward CCTCATGGCGCTTTTGTTGAC, reverse TCTGGTTCTGGGTGATGTTGA (PrimerBank ID 26787977c1); *sST2*: forward GAAGGCACACCGTAAGACTA, reverse GACAAACCAACGATAGGAGG (16); *ST2L*: forward GAAGGCACACCGTAAGACTA, reverse TTGTAGTTCCGTGGGTAGA (16); *GAPDH*: forward GAAGGTGAAGGTCGGAGTCA, reverse GACAAGCTTCCCGTTCTCA (17).

Quantitative real-time PCR was done using the PowerUp™ SYBR™ Green Master Mix (Applied Biosystems) in a final volume of 12 µl containing 0.5 µM of each primer using the QuantStudio 6 Flex (Applied Biosystems). The cycling conditions were as follows: 95°C for 10 min followed by 45 cycles of 95°C for 4 sec, 56°C for 5 sec, 72°;C for 15 sec. Experiments were performed in triplicates and quantification was performed using the 2^-ddCt^ method.

### Statistical analyses

Multiplex cytokine data was reported as concentration and for donor compilation, the data was transformed to logarithm scale which is more natural due to variation in concentration ranges between individual donors. The data was then scaled by subtracting the mean and dividing by standard deviation. These Z-scores are reported for each cytokine as described by Dingle et al. and were generated to account for variance of scale between individual donors (18). Results were analyzed on GraphPad Prism software (San Diego, CA). Ratio paired t tests were performed on qPCR experiments and paired t test were performed on Z-scores.

## Results

### Human differentiated eosinophils resemble mature eosinophils

**Figure 1:**
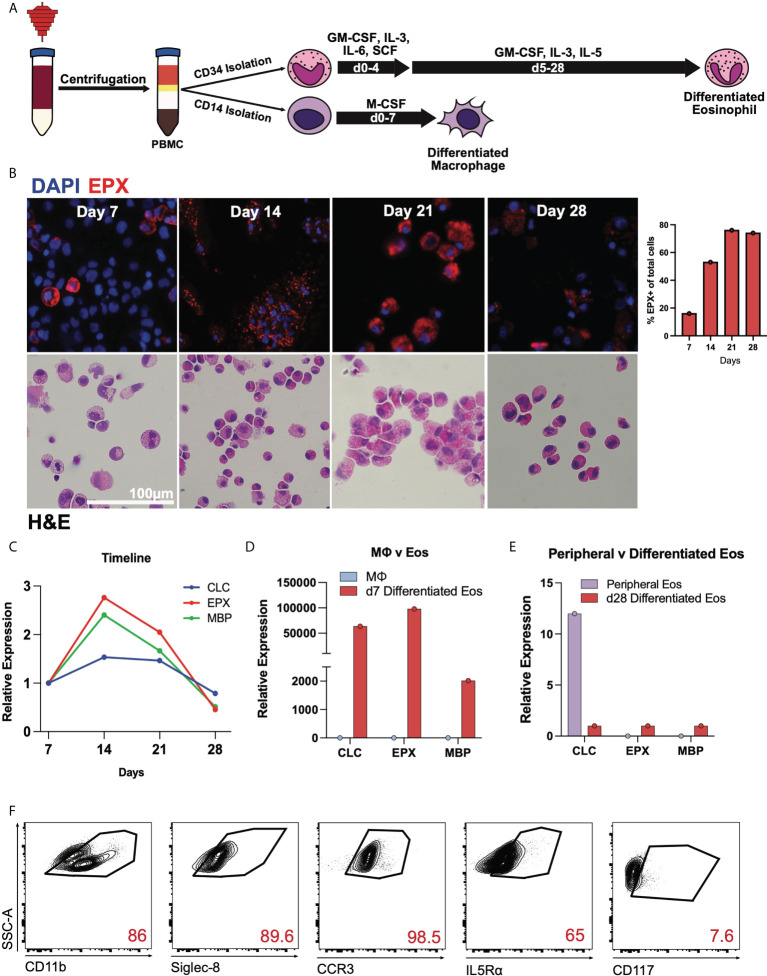
Human CD34+ hematopoietic stem cells mature into differentiated eosinophils. **(A)** Schema for differentiating eosinophis and macrophages from apheresis cones. **(B)** DAPI, EPX (above) and H&E staining of CD34+ cells during differentiation on days 7, 14, 21, and 28. Percent EPX+ cells of total cells shown graphically. **(C)** Expression of *CLC, EPX*, and *MBP* transcripts during differentiation relative to day 7 (baseline). **(D)** Expression levels of *CLC, EPX*, and *MBP* on day 7, relative to CD14+ cells. **(E)** Expression levels of *CLC*, *EPX* and *MBP* on mature peripheral eosinophils and day 28 differentiated eosinophils. **(F)** Flow cytometry of day 21 differentiated eosinophils for canonical eosinophil markers.

Using growth cytokine media, CD34+ hematopoietic stem cells were matured into differentiated eosinophils while CD14+ monocytes were differentiated into macrophages as indicated (**Figure 1A**). Throughout the differentiation process, cells were stained with hematoxylin and eosin at weekly timepoints demonstrating increasing eosinophilic appearance of these cells (**Figure 1B**). Similarly, immunofluorescence staining of eosinophil peroxidase (EPX) and DAPI nuclear dye identified EPX-containing eosinophil granules with percent EPX+ cells of total at differentiation timepoints shown (**Figure 1B**). Using RT-qPCR, we identified that canonical eosinophil markers including charcot-leyden crystal (*CLC*), *EPX* and major basic protein (*MBP*), are expressed by differentiated eosinophils (**Figure 1C**), the latter two of which are highly specific for eosinophils. Compared to day 7 cells, days 14 and 21 displayed the highest relative expression of these transcripts. When normalized to macrophages, day 7 differentiated eosinophils expressed high relative expression of *CLC*, *EPX* and *MBP* (**Figure 1D**). Expectedly, day 28 differentiated eosinophils and mature peripheral eosinophils in general have low transcriptional activity with *CLC* being the most abundant transcript in mature eosinophils (**Figure 1E**). As cells appeared to have the most robust expression of granule protein transcripts prior to day 21, subsequent experiments focused predominantly on these earlier timepoints. Flow cytometric analyses of day 21 differentiated eosinophils is notable for high expression of CD11b, CCR3, Siglec-8, and IL5Rα (**Figure 1F**). Gating strategy established based on unstained controls (**Supplemental Figure 1**).

### ST2L, sST2, IL-4, and IL-13 transcripts are expressed in human differentiated eosinophils

**Figure 2:**
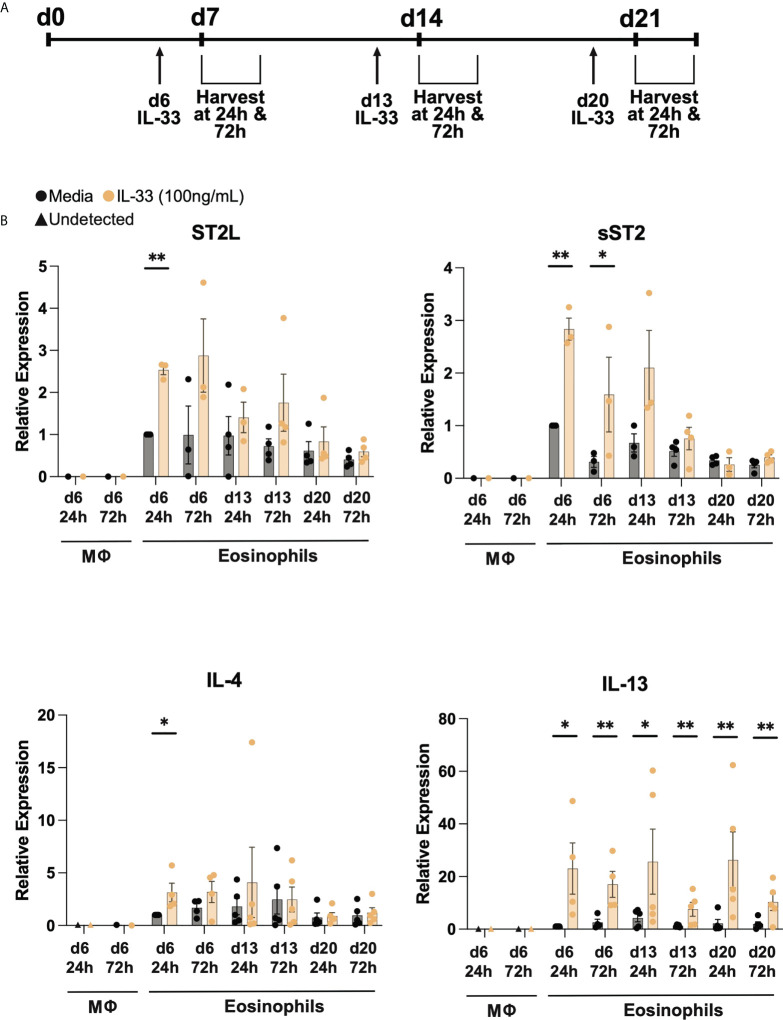
Differentiated eosinophils express IL33 receptor and type 2 cytokine transcripts. **(A)** IL-33 stimulation schema during differentiation. Cells were treated with IL-33 or media control on days 6, 13, and 20 (d6, d13, d20) and subsequently harvested 24h and 72h after each stimulation. **(B)** Relative expression levels of *ST2L, sST2, IL-4*, and *IL-13* using d6 24h media eosinophils as the control for each qRT-PCR. P < 0.05 (*), <0.0021 (**).

After validation of the eosinophil differentiation protocol, CD34+ or CD14+ cells were stimulated with or without 100 ng/mL IL-33 at designated timepoints and cells and supernatants were harvested at 24- and 72-hours post administration (**Figure 2A**). As noted in **Figure 1A**, macrophage differentiation concludes after one week of culture and therefore later timepoints were not examined. We first performed RT-qPCR to assess the relative expression of membrane-bound *ST2L*, *sST2* decoy receptor, *IL-4*, and *IL-13* at varied timepoints post stimulation (**Figure 2B**). We detected expression of all genes at all examined timepoints in eosinophils, regardless of IL-33 stimulation, but detected little if any expression of these receptors or cytokines in differentiated macrophages. The media control cells expressed *ST2L* and *sST2* maximally at d7 and then showed a slight decrease in expression as time went on. IL-33 stimulation increased *ST2L and sST2* expression in differentiating eosinophils, most notably when stimulated on days 6 and 13. However, for both *ST2L* and *sST2*, IL-33 had less effect 24h and 72h post day 20 stimulation. *IL-4* and *IL-13* transcripts in media control cells had the highest relative expression during the second week of the differentiation at day 13 72h post IL-33 and day 13 24h post IL-33, respectively. *IL-4* expression significantly increased in response to IL-33 24h and 72h post day 6 stimulation and then saw fewer changes after day 14. In contrast, *IL-13* expression was consistently elevated compared to media controls in response to IL-33 at all timepoints.

### IL-33 induces release of IL-4 and IL-13 from differentiated eosinophils

**Figure 3:**
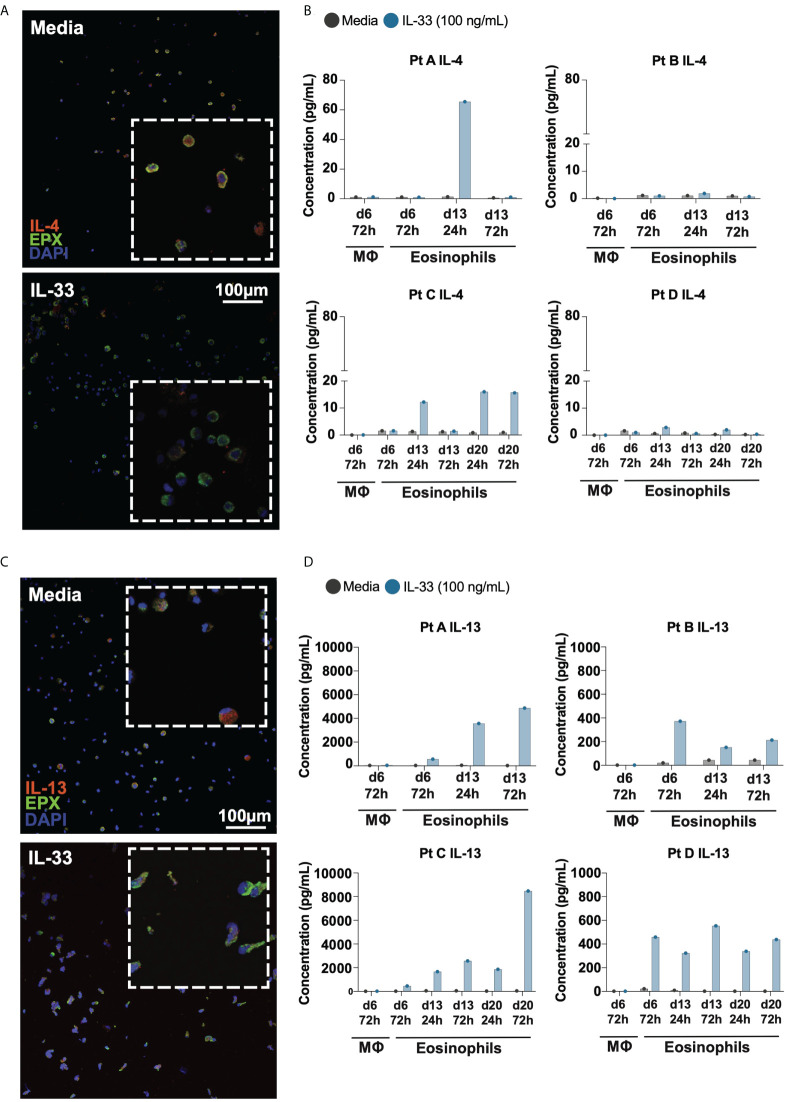
Differentiated eosinophils release IL-4 and IL-13 protein in response to IL-33. Immunofluorescence staining of differentiating eosinophils on d14 for **(A)** IL-4 and **(C)** IL-13. Cytokine analysis of supernatants of media and IL-33 treated cells at various time points detects **(B)** IL-4 and **(D)** IL-13 concentrations for each individual donor.

To determine whether IL-4 and IL-13 protein is expressed by and secreted from differentiated eosinophils, we performed immunofluorescence and Luminex multiplex cytokine analyses on the supernatants of differentiated eosinophils from four donor samples (**Figure 3**). Immunofluorescence of IL-4 revealed punctate granule-like staining intracellularly in cells treated with media; cells treated with IL-33 appeared to have less IL-4 intracellularly (**Figure 3A**). To assess IL-33’s ability to stimulate downstream cytokine secretion, we measured IL-4 concentration in the supernatant of differentiated eosinophils and found that secreted IL-4 generally increased in response to IL-33 24hrs after stimulation; this effect appeared to wane by 72hrs post-stimulation and was not consistently elevated across donor samples (**Figure 3B**). Similarly, IL-13 was robustly present in differentiated eosinophils kept in control media, as shown by immunofluorescence (Fgiure 3C). Treatment with IL-33 caused most eosinophils to secrete their IL-13 as measured by loss of immunofluorescent staining and increase in IL-13 protein in the culture media (**Figures 3C, D**). IL-13 accumulated to a greater concentration in the culture media than IL-4, and appeared to cumulatively increase from 24hr to 72hrs post-stimulation with IL-33 in all donors and at most differentiation timepoints (**Figure 3D**).

### IL-33 induces release of IL-13 and a wide array of chemokines

**Figure 4:**
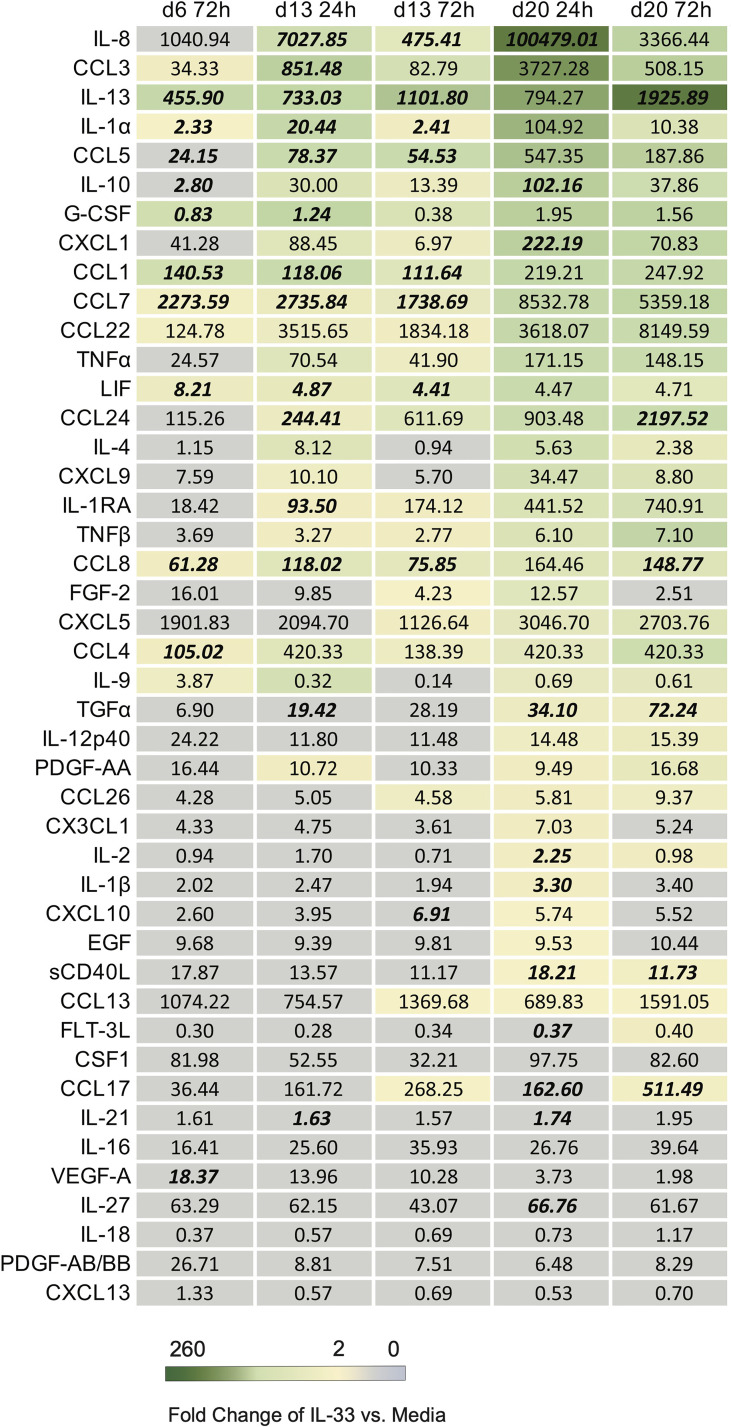
List of downstream cytokines produced in response to IL-33 in differentiated eosinophils. The geometric mean concentration of each cytokine in the IL-33 treated condition is shown numerically. A color gradient displays the level of fold change in concentration of IL-33 treatment relative to media control. Any cytokine with a fold change greater or equal to 2 was assigned color. Cytokines with a fold change less than 2 were left grey. The concentrations of cytokine from each donor and stimulus condition were transformed and plotted as Z-scores to account for varied scale between individuals. Cytokines at timepoints with statistically significant p values based on Z-scores in response to IL-33 compared to media control are bolded and italicized.

To assess what additional cytokines may be affected by IL-33 stimulus on differentiated eosinophils, the same cell supernatants that were examined for IL-4 and IL-13 were also examined using multiplex cytokine array at each timepoint (**Figure 4**). Geometric means of cytokine concentrations are shown, fold-change of IL-33 stimulated compared to media control, and Z-score p-values revealed IL-33 stimulated differentiated eosinophils significantly increased IL-13 secretion by both fold-change and Z-score p-value compared to control across almost all timepoints. At the timepoints measured during week 2 and week 3, we found consistent increases in fold change of IL-8, CCL3, CCL5, IL-13, IL-10, CXCL1, and CCL1 in the IL-33 treated group compared to media control. IL-8, CCL3 and IL-13 were secreted at high concentrations (**Figure 4**; see **Supplemental Table 1** for average concentrations of cytokines and fold change at given timepoints).

**Figure 5:**
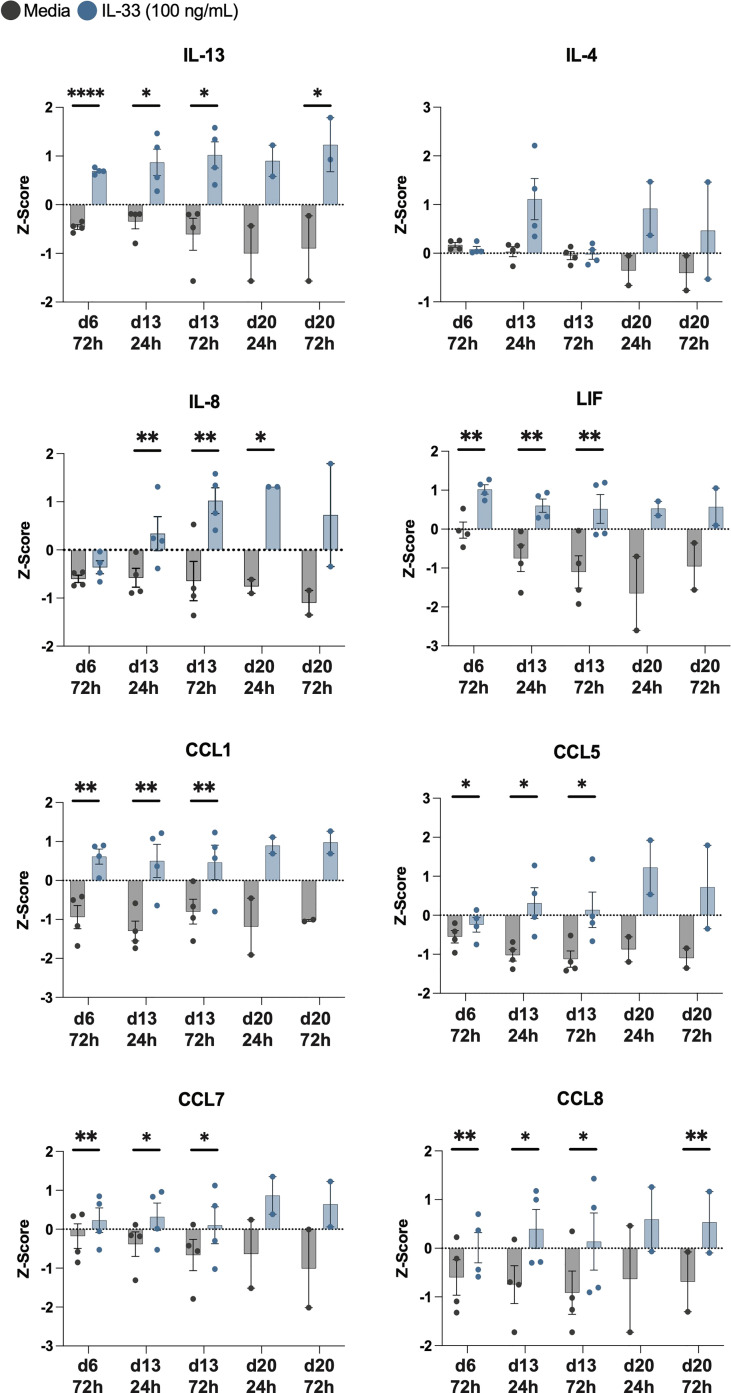
IL-33 significantly increases release of IL-13 among other cytokines from differentiated eosinophils. Z-scores of cytokines consistently upregulated by IL-33 treatment compared to media controls are displayed in addition to IL-4. P<0.05 (*), <0.0021 (**), <0.0001 (****).

Z-scores for each timepoint are shown for IL-13, IL-4, IL-8, LIF, CCL1, CCL5, CCL7, and CCL8 (**Figure 5**). Upon IL-33 treatment, CCL1, LIF, CCL7, and CCL5 were all significantly upregulated after IL-33 stimulation on day 6 and day 13, and trended upwards after day 20 stimulation. IL-4 did not reach statistical significance with IL-33 stimulation, but trended upwards 24h after each stimulation rather than at 72h post IL-33. IL-8 was significantly elevated in the IL-33 treated group 24h and 72h after the day 13 stimulation and 24h after the day 20 stimulation. IL-13 and CCL8 were significantly higher upon IL-33 treatment at almost every timepoint except for 24h after day 20, when it trended upwards.

## Discussion

Here we validated and detailed an approach to eosinophil differentiation from CD34+ hematopoietic stem cells and found that differentiated eosinophils directly respond to IL-33 with robust release of IL-13. In validating these cells, we showed that they are morphologically akin to peripheral eosinophils and express canonical eosinophil markers at the transcript and protein levels. IL-13 was secreted in large quantities in response to IL-33 across most timepoints of differentiation. Although we could show by immunofluorescence that IL-4 was released by eosinophils in response to IL-33, the absolute concentrations of IL-4 did not accumulate in culture media to high enough levels to reach statistical significance. Additional notable cytokines and chemokines that were secreted by eosinophils in response to IL-33 include IL-8, LIF, CCL1, CCL5, CCL7, and CCL8.

The differentiation of hematopoietic cells from CD34+ stem cells has been used for many hematopoietic cell lineages and was first described by Rosenberg et al. in 1996 for the differentiation of eosinophils and adapted by others in subsequent years (14, 19). Here, we provide an in-depth methodologic approach by adapting methods described by Rosenberg and conducted validation experiments showing that differentiated eosinophils are not only morphologically akin to naturally occurring eosinophils, but also express canonical eosinophil markers such as *EPX*, *MBP* and *CLC* and EPX protein similar to the protocol described by Legrand et al. (19). Keeping with findings shown by Legrand et al, these cells are most transcriptionally active between days 6-21 compared to day 28, a finding that was not surprising given that mature peripheral eosinophils are not considered to be particularly transcriptionally active and do not have long half-lives in circulations. Tissue-resident eosinophils are thought to have longer half-lives, although these cells have been difficult to study in humans given limited access to tissue compared to peripheral blood, and the lack of culture conditions capable of supporting eosinophil survival. Our studies here offer a window into longer-term culture conditions for eosinophils that may be applicable to studying the function of these cells in allergic disease as well as cancer and metabolic disease where tissue-resident eosinophils have also been shown to play a role (20, 21).

IL-33 is increasingly thought to be important for the pathogenesis of Th2 inflammatory responses and is a potential therapeutic target. Therefore, understanding its role in atopic diseases such as EoE are valuable undertaking. Previously, elucidating how eosinophils transcribe and release cytokines in response to IL-33 has been difficult due to the challenges of culturing mature eosinophils, particularly under conditions longer than 24-48 hours. Using our model, we detected ST2L as well as IL-4 and IL-13 transcripts as early as day 7, rendering these cells able to respond to IL-33 early in differentiation. Interestingly, we see that while all differentiating eosinophils show intracellular IL-4 and IL-13 by immunofluorescence (**Figures 3A, C**), only cells that received IL-33 secreted particularly IL-13 more so than IL-4 into supernatants, suggesting that IL-33 signaling *via* ST2 triggers selective release of cytokines (**Figures 3B, D**).

We found that differentiated eosinophils released IL-13 at both the 24h and 72h post-IL-33 timepoints with the latter time point having higher concentrations, while IL-4 detection was highest at 24h post-IL-33 stimulation and was only significant at one early timepoint. This difference may be due to the shorter half-life of IL-4 or to differences in the relative abundance of each cytokine in eosinophil granules. Importantly, we found that differentiated eosinophils secrete significant concentrations of IL-8 in response to IL-33, a finding that has been previously described using mature human peripheral blood eosinophils (22, 23) and which we were able to validate here. Additionally, IL-33 elicited significant concentrations of granulocyte-relevant chemokines such as CCL1, CCL5, CCL7, and CCL8 from differentiated eosinophils. CCL1 has been described previously as being important for chemotaxis of human monocytes and eosinophils (24, 25), and CCL5, CCL7, and CCL8 can elicit activating and chemotactic responses in eosinophils, CD4 T cells, and basophils (26–28). In a study of childhood asthma, CCL5 and CCL7 were significantly increased in the bronchial alveolar lavage (BAL) fluid of children with asthma and was associated with higher BAL eosinophils (29). Together, these findings suggest that IL-33 may elicit a specific cytokine signature that damages the surrounding tissue and further recruits other inflammatory cells.

Differentiating CD34+ hematopoietic stem cells into eosinophils should make mechanistic investigations of eosinophils more feasible. Studying these cells also has clinical relevance. In a prior study, CD34+ stem cells from blood donors express ST2L and produce IL-5 and IL-13 in response to IL-33 supporting these stem cells as potential effector cells in allergic diseases (30). They found IL-5 and IL-13 producing CD34+ stem cells were present in the sputum of patients with asthma, and increased after allergen challenge. Moreover, eosinophil progenitors, defined as CD34+ CD38+ and IL5Rα+ hematopoietic stem cells, were shown to be significantly elevated in the peripheral blood of patients with active EoE (31) and correlated with histologic disease activity (32). These findings further support the importance of studying eosinophil progenitors and the differentiation process in human cells.

Limitations of our study include application of this *in vitro* assay to tissue-resident or fully mature eosinophils. While we showed differentiated eosinophils express canonical markers of mature eosinophils, this was not 100% of the population even at late timepoints. Other important considerations include recognizing hematopoietic stem cells and EoP are likely more transcriptionally active than a fully mature eosinophil, which classically can be challenging to capture in this regard. This raises the importance of validating findings at the protein level.

In summary, we have validated and detailed an *in vitro* assay for differentiating human eosinophils from CD34+ hematopoietic stem cells and explored for the first time the effects of IL-33 signaling throughout the differentiation process. The ability of IL-33 to stimulate type 2 cytokine secretion, in combination with previous reports by others, strongly suggests that the IL-33-ST2 signaling pathway is an important axis for allergic responses. Further analyses of how IL-33 stimulated eosinophils may activate or affect migration of other leukocytes would reveal more about the immune interactions at sites of allergic inflammation.

## Data availability statement

The original contributions presented in the study are included in the article/**Supplementary Material**. Further inquiries can be directed to the corresponding author.

## Author contributions

AU and GR wrote the first draft of the manuscript and performed majority of the experiments. LQ conducted microscopy experiments. KP and JR provided intellectual guidance, equipment, reagents and lab space. MD and SD provided supervision of experiments, reagents and lab space and intellectual guidance. All authors contributed to the article and approved the submitted version.

## Funding

AU received funding through NIH T32DK007191 grant. LQ was funded by a SITC-Bristol-Myers Squibb Postdoctoral Cancer Immunotherapy Translational Fellowship. SD was funded by the Emerson Collective Cancer Research Fund, NIH U01 CA224146, NIH R01 AI158488-01, the Ludwig Center at Harvard and the Hale Family Center for Pancreatic Cancer Research. MD was funded by a Mentored Clinical Scientist Development Award 1K08DK114563.

## Acknowledgments

The authors also thank Paula Montero Llopis of the Harvard Medical School MicRoN core facility for microscopy assistance and the University of Utah Health Sciences Center Flow Cytometry Core Facility.

## Conflict of interest

AU does not have any conflicts related to this work but serves on the medical advisory board for Sanofi-Genzyme. MD receives research support from Novartis and Eli Lilly, consulting fees from Partner Therapeutics, Tillotts Pharma, ORIC Pharmaceuticals, AzurRx, SQZ, Moderna, Aditum, and Mallinckrodt, and is a member of the Scientific Advisory Board for Neoleukin Therapeutics. SD reports no conflicts of interest related to this study, but has received research support from Novartis, BMS, Genocea, and Eli Lilly, is a co-founder and SAB member for Kojin, and received consulting fees from GSK.

The remaining authors declare that the research was conducted in the absence of any commercial or financial relationships that could be construed as a potential conflict of interest.

## Publisher’s note

All claims expressed in this article are solely those of the authors and do not necessarily represent those of their affiliated organizations, or those of the publisher, the editors and the reviewers. Any product that may be evaluated in this article, or claim that may be made by its manufacturer, is not guaranteed or endorsed by the publisher.

## Supplementary material

Supplementary Figure 1 Flow cytometry gating strategy. Unstained control samples were used to establish gating parameters for differentiated eosinophils.
