# Supplementary figures and images for: Human differentiated eosinophils release IL-13 in response to IL-33 stimulation
Source: Front Immunol. 2022 Sep 13;13:946643. doi: 10.3389/fimmu.2022.946643 (PMC9513478; doi:10.3389/fimmu.2022.946643)

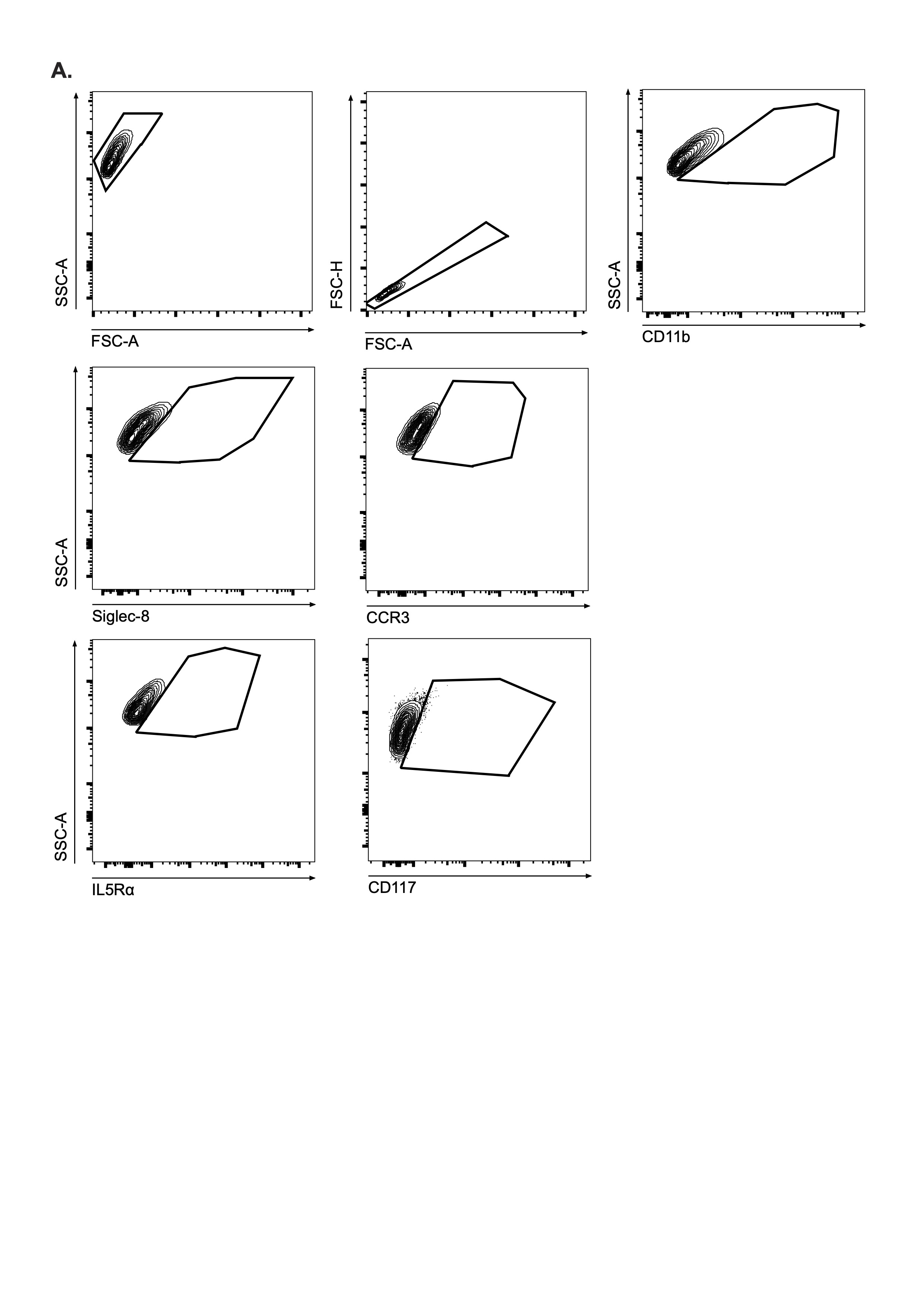

Supplement: Supplementary Figure 1 — Flow cytometry gating strategy. Unstained control samples were used to establish gating parameters for differentiated eosinophils. [file Image_1.jpg]
