# Supplementary material for: Human differentiated eosinophils release IL-13 in response to IL-33 stimulation
Source: Front Immunol. 2022 Sep 13;13:946643. doi: 10.3389/fimmu.2022.946643 (PMC9513478; doi:10.3389/fimmu.2022.946643)
Supplement: Supplementary file 2 [file Table_1.pdf]

Supplemental Table 1.

	d6 72h			d13 24h			d13 72h			d20 24h			d20 72h		
**Cytokine**	[Media]	[IL-33]	Fold Change	[Media]	[IL-33]	Fold Change	[Media]	[IL-33]	Fold Change	[Media]	[IL-33]	Fold Change	[Media]	[IL-33]	Fold Change
**CCL1**	16.43	157.91	9.61	9.27	190.42	20.55	22.08	180.23	8.16	14.62	231.08	15.81	10.88	273.27	25.13
**CCL13**	92.84	85.31	0.92	51.20	63.31	1.24	55.25	38.48	0.70	62.37	98.32	1.58	63.60	84.04	1.32
**CCL17**	36.12	39.71	1.10	134.38	206.60	1.54	158.21	354.88	2.24	96.64	171.67	1.78	220.89	517.55	2.34
**CCL22**	80.39	1660.48	20.66	10321.95	39824.74	3.86	1750.32	3593.89	2.05	215.18	3647.70	16.95	1022.14	9364.86	9.16
**CCL24**	145.32	144.43	0.99	92.18	302.31	3.28	137.57	1120.06	8.14	69.86	914.49	13.09	137.79	2264.91	16.44
**CCL26**	5.78	6.12	1.06	4.33	7.62	1.76	1.46	8.69	5.94	2.98	6.76	2.27	4.48	10.73	2.39
**CCL3**	15.50	53.10	3.43	40.27	1571.86	39.03	11.80	655.99	55.62	19.45	3739.78	192.33	12.48	1758.52	140.96
**CCL4**	44.97	139.17	3.09	133.90	420.33	3.14	69.09	187.68	2.72	87.62	420.33	4.80	53.57	420.33	7.85
**CCL5**	14.88	29.64	1.99	5.39	197.00	36.53	5.27	233.39	44.31	7.96	1256.26	157.92	4.57	912.83	199.96
**CCL7**	671.44	1117.81	1.66	592.10	799.31	1.35	864.99	1400.34	1.62	461.05	711.47	1.54	899.40	1594.51	1.77
**CCL8**	1652.45	3854.43	2.33	963.20	5051.59	5.24	715.37	5105.36	7.14	1198.18	13220.35	11.03	706.46	9798.22	13.87
**CSF1**	36.60	89.86	2.46	30.12	209.77	6.96	32.35	237.15	7.33	67.09	278.57	4.15	29.85	241.32	8.09
**CX3CL1**	3.50	6.17	1.76	2.78	5.72	2.06	2.78	4.06	1.46	2.78	10.27	3.69	2.78	6.32	2.27
**CXCL1**	34.47	56.09	1.63	19.31	209.90	10.87	1.78	44.07	24.72	6.69	279.61	41.80	2.90	352.00	121.38
**CXCL10**	3.68	2.83	0.77	2.93	4.19	1.43	4.17	8.23	1.97	3.27	5.87	1.80	3.25	5.95	1.83
**CXCL13**	1.29	1.50	1.16	0.44	0.58	1.34	0.69	0.73	1.07	0.74	0.56	0.76	0.67	0.73	1.09
**CXCL5**	11.88	12.19	1.03	10.93	11.88	1.09	10.15	12.43	1.22	10.05	12.11	1.21	10.29	12.64	1.23
**CXCL9**	56.69	32.93	0.58	5.58	13.77	2.47	14.70	22.95	1.56	24.25	41.20	1.70	3.74	15.59	4.17
**EGF**	2540.25	2267.13	0.89	1580.62	2801.33	1.77	515.83	2221.93	4.31	1169.66	3300.26	2.82	745.01	3194.87	4.29
**FGF-2**	14.76	16.50	1.12	14.27	10.46	0.73	5.08	7.76	1.53	4.52	12.71	2.81	7.87	8.26	1.05
**FLT-3L**	0.30	0.31	1.04	0.25	0.29	1.14	0.27	0.39	1.41	0.19	0.37	2.00	0.19	0.45	2.41
**G-CSF**	0.09	0.87	9.61	0.04	2.57	64.13	0.04	1.03	25.69	0.04	2.36	58.88	0.04	4.22	105.50
**IL-10**	1.23	2.40	1.96	1.04	58.13	56.03	1.06	3.73	3.53	0.92	117.48	128.39	2.64	15.25	5.77
**IL-12p40**	2.66	2.65	1.00	1.63	3.12	1.91	1.43	2.10	1.46	1.41	3.39	2.41	2.11	4.23	2.01
**IL-13**	32.66	38.47	1.18	51.03	168.40	3.30	87.93	452.86	5.15	59.70	459.02	7.69	101.17	774.55	7.66
**IL-16**	1.07	1.05	0.98	1.07	1.80	1.68	1.57	1.44	0.92	0.94	2.28	2.44	0.50	1.10	2.20
**IL-18**	1.38	1.17	0.85	1.10	20.61	18.74	0.93	0.99	1.06	0.59	9.00	15.38	0.56	8.08	14.43
**IL-1RA**	574.47	1271.08	2.21	911.48	27872.22	30.58	75.60	3102.14	41.03	378.20	100479.0	265.68	129.23	6929.11	53.62
**IL-1α**	1.38	4.46	3.24	0.03	0.57	19.08	0.29	0.36	1.23	0.35	0.70	2.01	0.21	0.63	3.05
**IL-1β**	2.57	3.33	1.30	3.86	94.07	24.40	4.21	29.66	7.05	2.15	124.35	57.97	3.56	63.42	17.84
**IL-2**	27.03	24.85	0.92	8.40	12.92	1.54	7.22	13.39	1.85	5.38	15.19	2.82	3.78	18.33	4.85
**IL-21**	21.61	460.73	21.32	34.44	1426.77	41.43	26.86	2050.12	76.34	11.89	1101.28	92.66	20.15	4452.81	220.98
**IL-27**	16.28	17.49	1.07	22.80	27.07	1.19	28.91	42.59	1.47	21.68	29.93	1.38	37.25	40.14	1.08
**IL-4**	0.42	0.46	1.09	0.54	0.65	1.20	0.61	0.85	1.39	0.69	0.87	1.26	1.10	1.17	1.07
**IL-8**	1.53	1.62	1.06	1.36	1.64	1.21	1.44	1.58	1.10	1.19	1.74	1.47	2.00	1.98	0.99
**IL-9**	63.69	63.45	1.00	55.48	62.64	1.13	42.36	48.91	1.15	57.03	66.95	1.17	55.16	61.82	1.12
**LIF**	2.48	8.48	3.43	1.14	5.21	4.57	0.88	5.91	6.73	0.53	4.59	8.73	0.91	5.57	6.15
**PDGF-AA**	15.87	17.59	1.11	4.84	13.74	2.84	7.72	17.40	2.25	5.74	12.00	2.09	3.76	28.17	7.50
**PDGF-AB/BB**	33.10	27.82	0.84	10.38	9.91	0.95	5.92	9.95	1.68	6.87	7.54	1.10	8.41	8.71	1.04
**sCD40L**	15.80	18.18	1.15	11.14	13.58	1.22	8.17	11.75	1.44	8.70	18.22	2.09	6.08	13.49	2.22
**TGFα**	8.02	8.38	1.04	13.28	28.39	2.14	21.44	51.07	2.38	8.67	34.86	4.02	22.53	88.44	3.93
**TNFα**	20.65	27.62	1.34	10.97	206.00	18.78	11.22	94.28	8.40	9.49	226.06	23.82	9.92	260.64	26.27
**TNFβ**	5.24	3.77	0.72	1.40	4.55	3.25	1.82	3.80	2.08	0.73	6.24	8.54	0.95	7.37	7.80
**VEGF-A**	48.34	24.34	0.50	34.69	25.39	0.73	68.06	30.84	0.45	4.79	6.74	1.41	25.57	2.30	0.09

[ ] = Average Concentration in pg/mL
Fold Change = [IL-33]/[Media]
